# Domain-specific functions of LRIT3 in synaptic assembly and retinal signal transmission

**DOI:** 10.1016/j.jbc.2026.113097

**Published:** 2026-04-27

**Authors:** Nazarul Hasan, Ronald G. Gregg

**Affiliations:** 1 Department of Biochemistry & Molecular Genetics, School of Medicine, University of Louisville, Louisville, Kentucky, USA; 2 Department of Ophthalmology & Visual Sciences, School of Medicine, University of Louisville, Louisville, Kentucky, USA

**Keywords:** retina, synapse, protein domain, protein complex, gene therapy, LRIT3, LRR proteins, congenital stationary night blindness, TRPM1, nyctalopin, photoreceptors, rAAV, transsynaptic interactions

## Abstract

LRIT3 is a leucine-rich repeat (LRR) protein that is expressed in the retina, and its absence causes complete congenital stationary night blindness (cCSNB), a genetically diverse disorder characterized by impaired low-light vision, myopia, and nystagmus. LRIT3 is expressed in rod and cone photoreceptors, and it transsynaptically organizes the assembly of the glutamate signaling complex, the signalplex, on depolarizing bipolar cells (DBCs). LRIT3 is a single-pass membrane protein with extracellular LRR, IG, and FN3 domains. We express domain deletion constructs using rAAV and examine the impact on LRIT3 trafficking, as well as the structural and functional recovery of the signalplex in DBCs. We show the LRR domain may be required for trafficking LRIT3 to the synapse in cones, but not rods, and it is needed for reassembly and function of the rod BC signalplex. The IG domain is required for the localization of TRPM1 to the signalplex and thus its function. The FN3 domain is not necessary for either DBC signalplex assembly or function. Our data demonstrate that the LRR and IG domains of LRIT3 are crucial for TRPM1 localization and retinal function, and that restoring Nyctalopin localization to the DBC signalplex alone is insufficient to restore TRPM1 expression. Based on our findings, we propose a model in which the LRR domain transsynaptically binds with Nyctalopin, while the IG domain interacts with TRPM1.

Vision begins in the retina with rod and cone photoreceptor cells, which are specialized for detecting light. When light levels increase, these photoreceptors hyperpolarize and reduce their release of glutamate in the outer plexiform layer (OPL). This change is detected by second order neurons, bipolar (BC) and horizontal cells. Of the 15 bipolar types (1), six hyperpolarizing bipolar cells (HBCs) in response to a light increment and nine depolarizing bipolar cells (DBCs). Rod photoreceptors primarily connect to a single type of DBC, while cones connect to eight types of DBCs and six types of HBCs. DBCs detect changes in glutamate release through the metabotropic glutamate receptor 6 (mGluR6) (2, 3), which modulates the nonspecific cation channel TRPM1 (4, 5, 6). Cones connect to two types of bipolar cells: DBCs, which also use mGluR6, and HBCs that use AMPA/kainate ionotropic glutamate receptors (7, 8, 9, 10). Rod bipolar cells and horizontal cells form a single invaginating synapse near a ribbon structure within the rod photoreceptor spherule. Similarly, cone pedicles have multiple invaginating synapses involving various classes of cone DBCs and horizontal cell dendrites, all positioned near a ribbon. In contrast, HBCs form flat contacts on the base of the cone pedicle. This intricate synaptic organization allows the retina to process and transmit visual information efficiently, enabling us to see in both dim and bright light conditions.

Anatomically, invaginating synapses of photoreceptors are intricate structures involving multiple presynaptic and postsynaptic proteins, including several adhesion molecules that maintain synapse stability and functionality (11, 12, 13, 14, 15). Despite knowing many of the involved components, the molecular mechanisms underlying synapse formation and receptor complex assembly remain poorly understood. Mutations in *GRM6*, *TRPM1*, *Nyctalopin*, *GPR179*, and *LRIT3* have been identified as disruptors of ON pathway signaling in humans, leading to complete congenital stationary night blindness (cCSNB, see review by Zhang *et al.* (16)). In mice, these have all been shown to be a part of a large signalplex located on the tips of the DBC dendrites.

LRIT3 is a leucine-rich repeat (LRR) protein that is involved in transsynaptic organization of the DBC signalplex and is a single-pass membrane protein that contains an extracellular LRR, immunoglobulin-like (IG), and fibronectin type III (FN3) domains. Three other LRR-containing proteins, Nyctalopin, ELFN1, and ELFN2, also are involved in synaptic protein assembly and/or synaptic transduction in the OPL. LRIT3 is expressed exclusively at rod and cone synapses with DBCs (12, 13, 17). Functionally, the loss of LRIT3 results in a no b-wave phenotype in mice (12, 13). In the absence of LRIT3, there are differential effects on rod and cone DBC signalplexes. In rods, Nyctalopin and TRPM1 fail to localize at rod BC dendrites, and in cones the entire postsynaptic signalplex, including mGluR6, GPR179, Nyctalopin, TRPM1, and RGS7/11 is missing (13, 18, 19). Given these differences between rod and cones synapses in *Lrit3*^*−/−*^ retinas, we hypothesized that the extracellular domains of LRIT3 contribute to its differential functions, with each domain playing a specific role in synaptic protein assembly.

We now demonstrate that the LRIT3 IG domain is crucial for the localization of TRPM1 at both rod and cone synapses, and the LRR domain is essential for the localization of Nyctalopin and TRPM1 at rod synapses.

## Results

**Figure 1:**
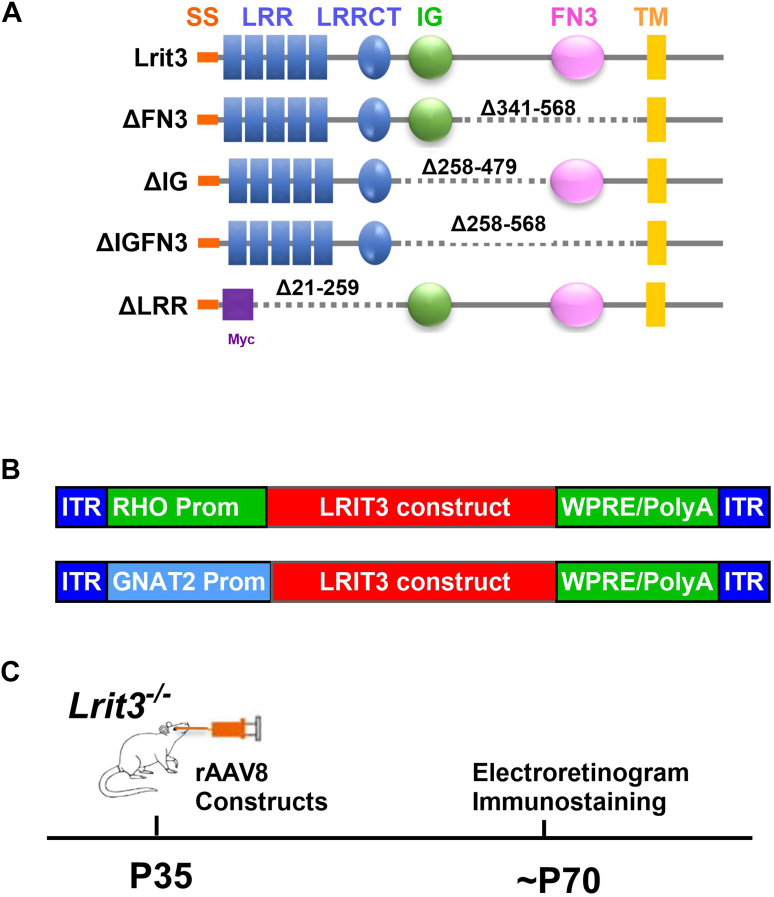
**Experimental design and vector constructs for LRIT3 expression in mouse retina.***A*, structure-based design of LRIT3 protein constructs with systematic deletion of extracellular domains. WT LRIT3 and four truncated variants with selective removal of fibronectin type III (FN3), immunoglobulin-like (IG), and leucine-rich repeat (LRR) domains, respectively. The numbers indicate the deleted amino acids (ENSMUST00000185462.6). Not to scale. *B*, schematic of the two recombinant adeno-associated virus (rAAV) vectors containing rhodopsin (RHO) and GNAT2 promoter for targeting rod and cone photoreceptors, respectively. *C*, experimental workflow: Adult (P35) *Lrit3*^−/−^ mice received subretinal rAAV injections. Thirty-five days after treatment, retinal function was assessed by electroretinography (ERG) and protein expression was evaluated by immunohistochemistry. SS, signal sequence; LRR and LRRCT, leucine-rich repeats; IG, Ig domain; FN, Fn3 domain; TM, transmembrane domain.

Mutations in the *LRIT3* gene have been identified as a cause of complete congenital stationary night blindness in humans (20) and the expression of full-length LRIT3 in cones or rods of *Lrit3*^*−/−*^ mice restores function (13, 17, 21). Because LRIT3 has differential effects on rod and cone synapses (12, 13) and has three predicted functional domains, LRR, IGc2 (IG), and FN3, we asked whether these specific domains have distinct functions. To explore this, we created deletion constructs lacking the LRR, IG, or FN3 domains, as well as both IG and FN3. (Fig. 1*A*). Because the LRIT3 antibody was generated against the LRR domain, we added a Myc tag after amino acid 19 in the ΔLRR constructs to allow immunohistochemistry (IHC). To limit expression to either rods or cones, we used the rhodopsin (RHO) promoter (22) or the GNAT2 promoter (23), respectively. Figure 1*B* shows the general design of the adeno-associated virus (AAV) constructs packaged into rAAV8 capsids. The recombinant adeno-associated viruses (rAAVs) were then injected into the subretinal space of *Lrit3*^*−/−*^ mice on postnatal day ∼35 (P35), and protein trafficking and function were assessed at ∼ P70, 5 weeks post-injection (Fig. 1*C*). To visualize Nyctalopin, we crossed an EYFP-tagged Nyctalopin transgenic line (24), onto the *Lrit3*^*−/−*^ mice and stained with anti-GFP antibodies, and we refer to this as YFP-Nyx. The complementary DNA (cDNA) sequences of all deletion constructs are provided in Table S1.

### LRIT3 domain requirements for DBC signalplex assembly and function

**Figure 2:**
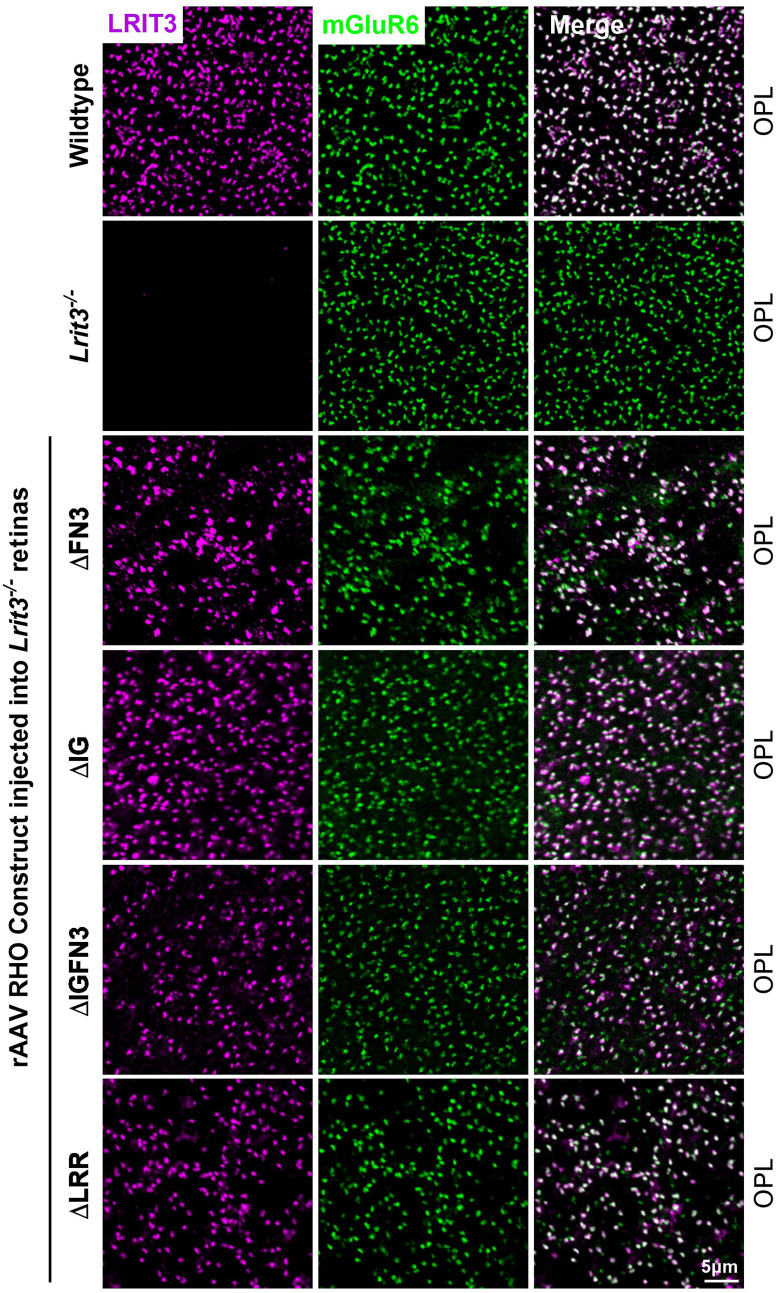
**LRIT3 deletion constructs successfully traffic and localize at rod synapses.** Representative confocal images of whole mount retinas from *Lrit3*^*−/−*^ and WT mice and *Lrit3*^*−/−*^ mice treated with rAAV8 RHO::ΔFN3, rAAV8 RHO::ΔIG, rAAV8 RHO::ΔIGFN3, and rAAV8 RHO::ΔLRR. Images were captured as maximum projections of z-stacks acquired at the OPL after staining with antibodies against LRIT3 (*magenta*) and mGluR6 (*green*). The scale bar represents 5 μm. OPL, outer plexiform layer; mGluR6, metabotropic glutamate receptor 6; rAAV8, recombinant adeno-associated virus type 8.

**Figure 3:**
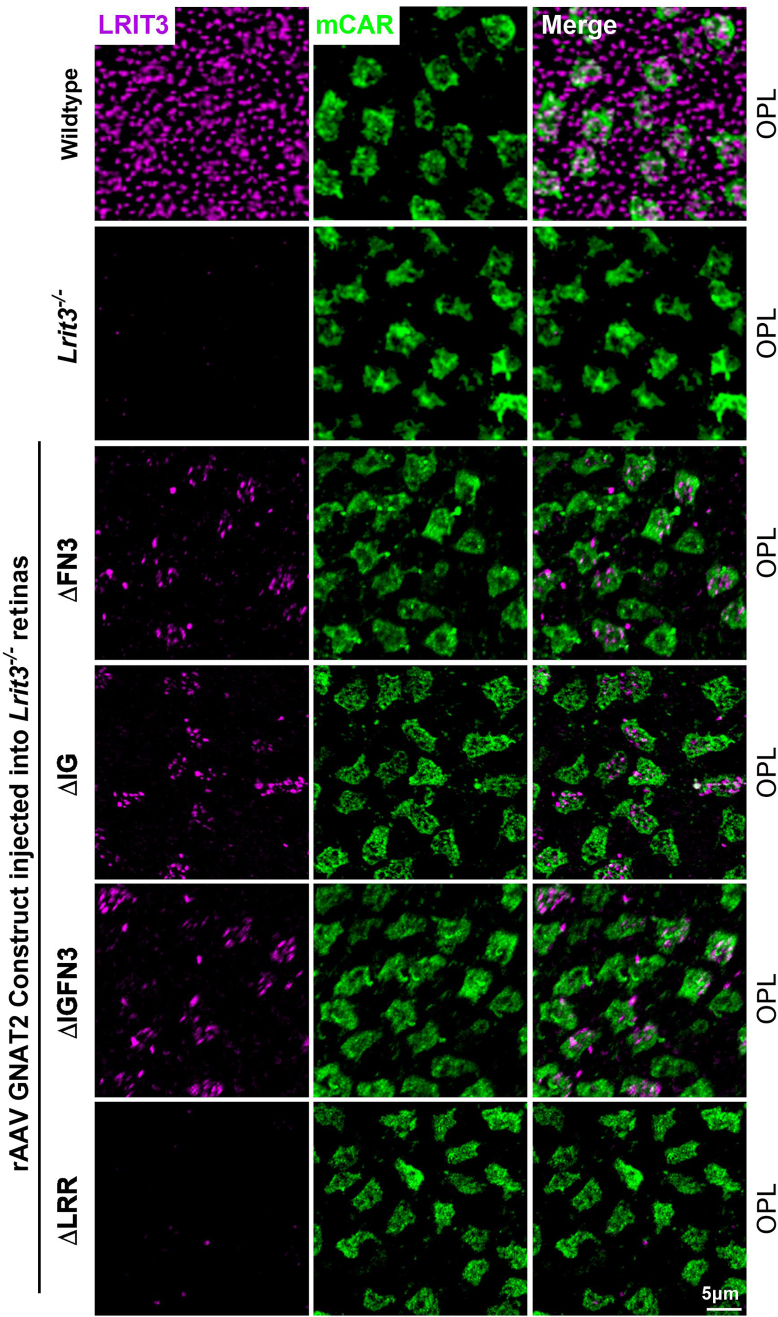
**Most LRIT3 deletion constructs traffic successfully and localize at cone synapses.** Representative confocal images of whole mount retinas from *Lrit3*^*−/−*^ and WT mice, and *Lrit3*^−/−^ mice treated with rAAV8 vectors expressing GNAT2-driven LRIT3 deletion constructs (ΔFN3, ΔIG, ΔIGFN3, and ΔLRR). Images were acquired at the OPL level after immunostaining with antibodies against LRIT3 (*magenta*) and mouse cone arrestin (mCAR, *green*). The scale bar represents 5 μm. OPL, outer plexiform layer; rAAV8, recombinant adeno-associated virus type 8.

**Figure 4:**
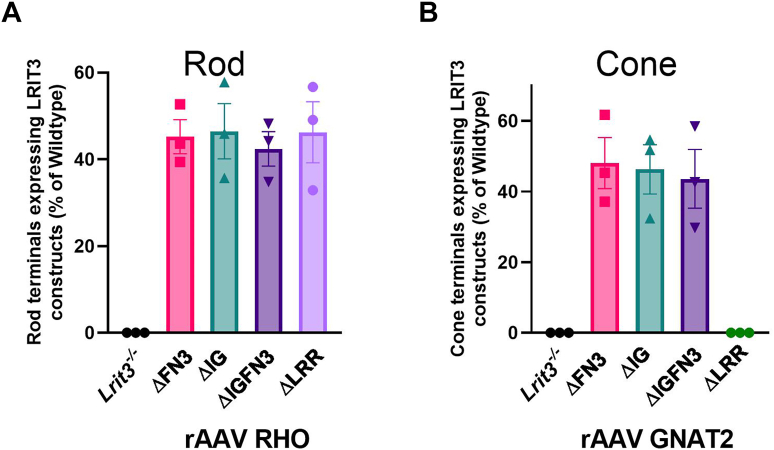
**Most LRIT3 deletion constructs traffic to rod and cone synapses.***A*, quantitative analysis of LRIT3 deletion construct expression at rod synapses. *Bar graph* shows the percentage of rod synapses positive for each deletion construct in *Lrit3*^*−/−*^ retinas (n = 3 eyes from three mice per group). *B*, quantitative analysis of LRIT3 deletion constructs expression at cone synapses. *Bar graph* displays the percentage of cone synapses positive for each deletion construct in *Lrit3*^−/−^ retinas following treatment (n = 3 eyes from 3 mice per group). OPL, outer plexiform layer.

Expression of deletion constructs used to probe the function of various protein domains in LRIT3 may lead to abnormalities in protein trafficking. To address this, we used IHC to determine if trafficking was normal and whether other components known to be absent in *Lrit3*^*−/−*^ mice (13, 18, 21) were relocalized to the rod and cone DBC signalplexes. We also did western blots on a small number of retinas treated with the rAAV RHO deletion constructs and showed the altered size of the LRIT3 protein was as expected (Fig. S1). To examine expression and quantify the infection frequency and trafficking in retinas injected with rAAVs expressing mutant forms of LRIT3 in rods or cones, we immunostained whole mount retinas. We used colocalization of LRIT3 with mGluR6 in rods (Fig. 2) and with cone arrestin (mCAR) in cones (Fig. 3) in the OPL, to assess trafficking and the fraction of the cells showing expression of the various constructs (Fig. 4). Example images for WT, *Lrit3*^*−/−*^ and *Lrit3*^*−/−*^ treated with the deletion constructs, are shown for a small section of a whole mount retina for each (Figs. 2 and 3). For quantification, colocalization of staining in the OPL from five regions of each retina/construct in at least three separate retinas was imaged, and the fraction of rods and cones terminals expressing the LRIT3 constructs was quantified (Fig. 4).

**Figure 5:**
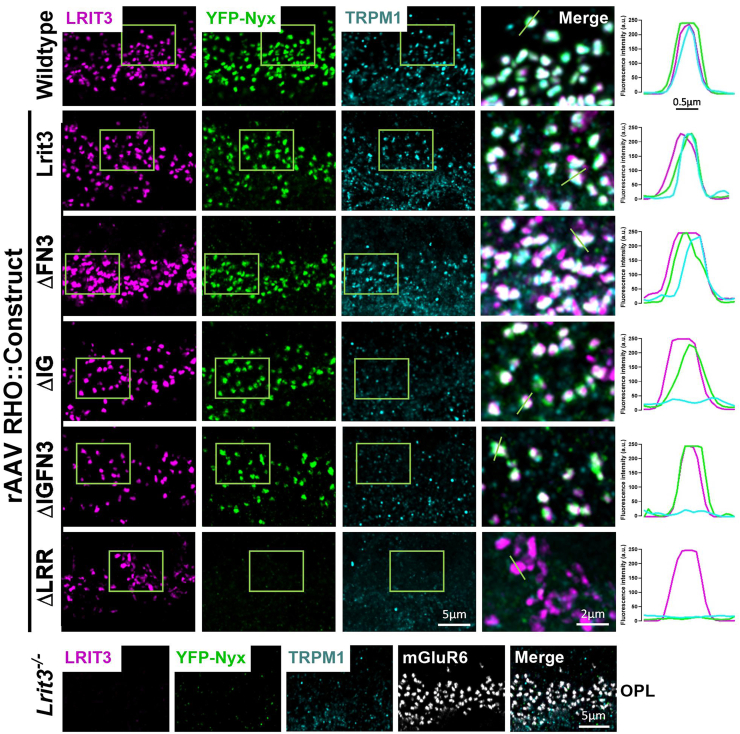
**LRR and IG domains of LRIT3 are essential for TRPM1 localization at rod bipolar cell dendrites.** Representative confocal images of transverse sections of the OPL from WT and *Lrit3*^*−/−*^ retinas treated with rAAV8 vectors expressing RHO-driven constructs: full-length Lrit3, ΔFN3, ΔIG, ΔIGFN3, or ΔLRR. Mice also express the YFP-Nyx transgene. Sections were immunostained using antibodies to LRIT3 (*magenta*), GFP (YFP-Nyx, *green*), and TRPM1 (*cyan*). The scale bar represents 5 μm. *Boxed* regions are shown as enlarged merged images from each condition. The scale bar represents 2 μm. Images are representative of n = 4 retinas per group. Quantitative intensity profile analysis (*right* panels) shows the spatial distribution and colocalization of LRIT3, Nyctalopin, and TRPM1 at rod synaptic terminals, measured along the designated *green line* in the merged images. *Bottom* row: staining of a *Lrit3*^*−/−*^ retina for mGluR6, LRIT3 TRPM1, and YFP-Nyx, all of which are lost except mGluR6 at rod synapses. OPL, outer plexiform layer; LRR, leucine-rich repeat; IG, immunoglobulin; RHO, rhodopsin; mGluR6, metabotropic glutamate receptor 6; rAAV8, recombinant adeno-associated virus type 8.

**Figure 6:**
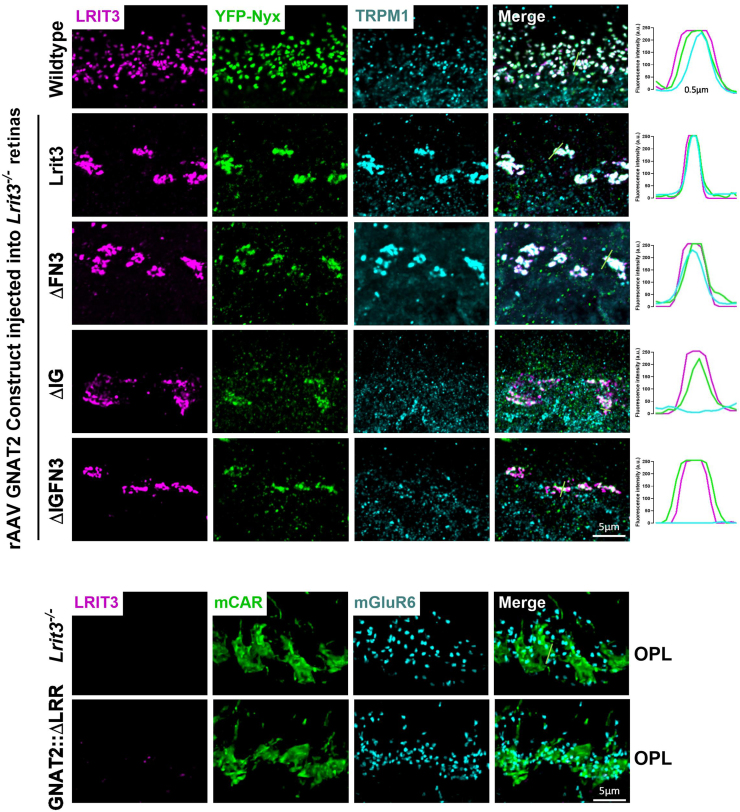
**LRR domain of LRIT3 is essential for localization of LRIT3, nyctalopin, and TRPM1 at cone synapses.** Representative confocal images of transverse retinal sections of the OPL from WT and *Lrit3*^−/−^ retinas following subretinal delivery of rAAV8 vectors containing GNAT2 promoter-driven constructs: full-length Lrit3, ΔFN3, ΔIG, ΔIGFN3, and ΔLRR deletion mutants. Mice also express the YFP-Nyx transgene. Triple immunostaining for LRIT3 (*magenta*), GFP (YFP-Nyx, *green*), and TRPM1 (*cyan*). The scale bar represents 5 μm. Images are representative of four independent retinas per experimental group. Quantitative intensity profile analysis (*right* panels) shows the spatial distribution and colocalization of Nyctalopin, TRPM1, and LRIT3 at cone synaptic terminals, measured along the designated *green line* in the merged images. *Bottom panels* show staining in *Lrit3*^−/−^ retinas and those transfected with the ΔLRR construct. OPL, outer plexiform layer; rAAV8, recombinant adeno-associated virus type 8; LRR, leucine-rich repeat. Note: The WT panel is replicated from Figure 5.

To examine the impact of the various deletions on signalplex assembly and colocalization, we used IHC to triple-label transverse retinal sections for LRIT3, Nyctalopin (YFP-Nyx-EYFP), and TRPM1 for rod (Fig. 5) and cone (Fig. 6) specific constructs. We also created intensity plots across puncta for each of the three antibodies (graphs to the right of the images in Figs. 5 and 6), which showed that the deletion constructs trafficked and colocalized with their known postsynaptic partners.

When full-length LRIT3 is expressed in rods of *Lrit3*^*−/−*^ mice, it traffics and restores DBC signalplex assembly and function (21). When the deletion constructs are expressed in rods, they all traffick as is evident from the punctate labeling seen for LRIT3 that colocalizes with mGluR6, in the OPL (Fig. 2). In rods, except for the ΔLRR construct Nyctalopin expression (YFP-Nyx) is restored to the OPL as indicated by its colocalization with LRIT3 and mGluR6 for the ΔFN3, ΔIG, and ΔIGFN3 constructs (Fig. 5). Thus, the LRR domain is essential for Nyctalopin's correct localization. For correct localization of TRPM1, the IG and LRR domains are required, because it is not localized correctly when *Lrit3*^*−/−*^ eyes are treated with either the ΔIG or ΔLRR constructs. Because Nyctalopin is restored when the ΔIG and ΔIGFN3 constructs are expressed but TRPM1 is not, this indicates our earlier hypothesis that Nyctalopin was sufficient to localize TRPM1 was incorrect (11). Rather, Nyctalopin and LRIT3 are both required for TRPM1 localization to rod BC synapses.

To examine the function of LRIT3 domains on cone DBC signalplex assembly, we treated *Lrit3*^*−/−*^ retinas with rAAV using the GNAT2 promoter to drive expression of domain deletion constructs (Figs. 3 and 6). We then immunostained transverse retinal sections for LRIT3, Nyctalopin (YFP-Nyx), and TRPM1 (Fig. 6). These images are representative of at least four separate retinas. The ΔIG and/or the ΔFN3 localized to the OPL correctly, but unlike in rods, the ΔLRR domain construct did not traffic to the correct location on cone pedicles, so its function in assembling the cone DBC signalplex cannot be determined. Careful examination of transverse retinal sections from multiple retinas failed to detect any ΔLRR LRIT3 protein anywhere in the cones, suggesting it underwent rapid degradation. Like the situation in rods, the IG and/or the FN3 domain was not needed for localization of ΔLRIT3s to the cone pedicle (Fig. 6), and the FN3 domain is not required for Nyctalopin localization. For TRPM1 localization, the IG domain was needed, based on a failure of the ΔIG and the ΔIGFN3 (Fig. 6) constructs to localize TRPM1 to the OPL. As in the rod pedicles, Nyctalopin localization is insufficient to restore TRPM1 to the signalplex of cone DBCs (Fig. 6). Trafficking in rods and cones appears to have different criteria, as the ΔLRR construct trafficked normally in rods but not in cones. The ΔLRR construct also includes a twenty-four amino acid linker sequence containing the Myc-tag, required for IHC. Therefore, it is possible that this tag interferes with trafficking in cones. We suspect this is not the case because knock-in of a Myc-tagged BioID between codons for amino acids 19 and 20 of *LRIT3* gene in mice, or insertion of APEX into a full-length LRIT3 AAV construct did not disrupt LRIT3 function (Figs. S2 and S3). Thus, the exact reason the ΔLRR construct traffics in rods but not cones remains to be determined.

**Figure 7:**
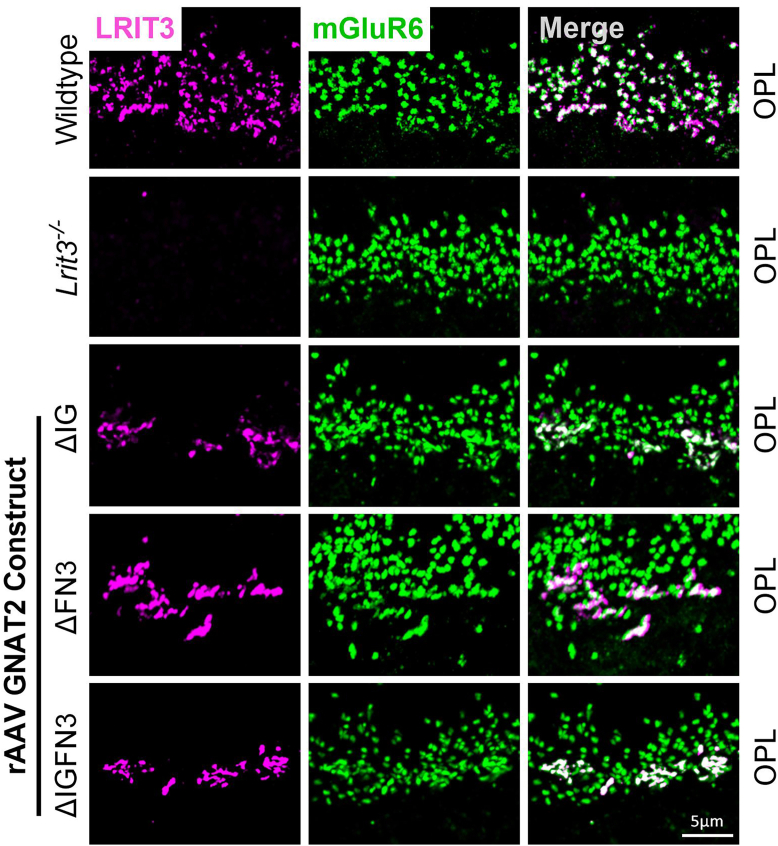
**mGluR6 localizes at cone bipolar cell dendrites in the absence of LRIT3 IG and FN3 domains.** Representative confocal images of transverse retinal sections from the OPL of WT, *Lrit3*^−/−^, and *Lrit3*^−/−^ retinas treated with subretinal injections of rAAV8 vectors expressing GNAT2-driven LRIT3 deletion constructs: ΔIG, ΔFN3, and ΔIGFN3. Sections were immunostained with antibodies against LRIT3 (*magenta*) and mGluR6 (*green*). The scale bar represents 5 μm. Images are representative of n = 4 retinas per group. OPL, outer plexiform layer; FN3, fibronectin type III; mGluR6, metabotropic glutamate receptor 6. IG, immunoglobulin; rAAV8, recombinant adeno-associated virus type 8.

**Figure 8:**
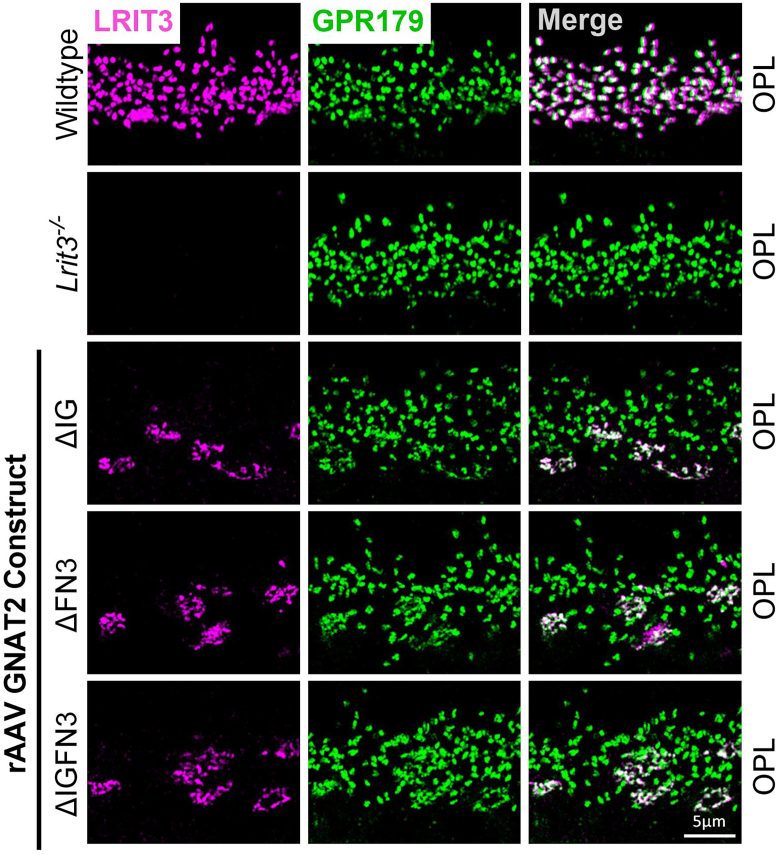
**GPR179 localizes at cone bipolar cell dendrites in the absence of LRIT3 IG and FN3 domains.** Representative confocal images of transverse retinal sections from the OPL of WT mice, *Lrit3*^−/−^, and *Lrit3*^−/−^ retinas treated with subretinal injections of rAAV8 vectors carrying GNAT2-controlled LRIT3 constructs lacking specific domains: ΔIG, ΔFN3, and ΔIGFN3. Sections were immunostained with antibodies against LRIT3 (*magenta*) and GPR179 (*green*). The scale bar represents 5 μm. Images are representative of n = 4 retinas per group. The scale bar represents 5 μm. OPL = outer plexiform layer. FN3, fibronectin type III; rAAV8, recombinant adeno-associated virus type 8.

In *Lrit3*^*−/−*^ mice, mGluR6 and GPR179 are also missing from the dendrites of cone DBCs, but present on the rod BC dendrites (Figs. 7 and 8, (13, 17, 21)). Therefore, we examined their localization after treatment of *Lrit3*^*−/−*^ mice with cone specific GNAT2::ΔFN3, ΔIG and ΔIGFN3 constructs. In all cases, mGluR6 (Fig. 7) and GPR179 (Fig. 8) were restored to the cone DBC dendrites. Note that for both mGluR6 and GPR179, the small green puncta in the merged image represents their presence on rod BC dendrites, which do not require LRIT3 for its localization in the *Lrit3*^*−/−*^ retinas. Whether GPR179 and mGluR6 require the LRR domain for localization in cone DBCs remains unknown, because the ΔLRR construct failed to traffic to cone synapses (Fig. 6).

### The LRIT3 FN3 domain is not required for DBC function

**Figure 9:**
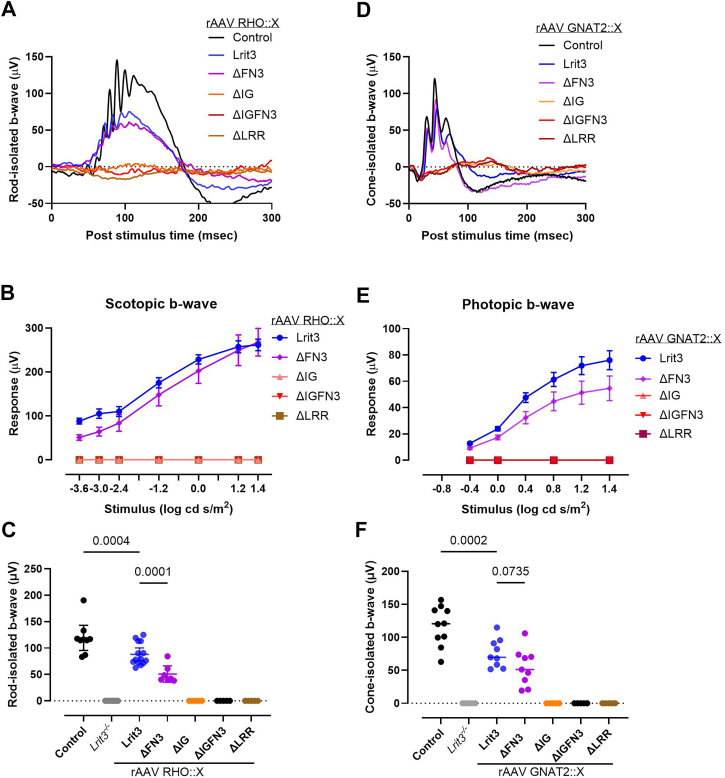
**LRR** and **IGdomains of LRIT3 are critical for synaptic transmission and retinal function.***A*, representative rod-isolated scotopic full-field electroretinogram (ffERG) waveforms recorded at −3.6 log cd s/m^2^ from control mice (WT treated with rAAV8 GRK1::GFP, n = 9), *Lrit3*^−/−^ mice (n = 8), and *Lrit3*^−/−^ mice treated with rAAV8 RHO-driven constructs: full-length Lrit3 (n = 11), ΔFN3 (n = 7), ΔIG (n = 7), ΔIGFN3 (n = 7), and ΔLRR (n = 7). *B*, quantitative analysis of scotopic ffERG b-wave amplitudes across multiple flash intensities for experimental groups described in *panel A*. *C*, rod-isolated b-wave amplitudes from scotopic ffERG recordings at −3.6 log cd s/m^2^ comparing all treatment groups from *panel A*. *D*, representative cone-isolated photopic ffERG waveforms recorded at 1.4 log cd s/m^2^ from control mice (WT treated with rAAV8 GRK1::GFP (n = 9), *Lrit3*^−/−^ mice (n = 8), and *Lrit3*^−/−^ mice treated with rAAV8 GNAT2-driven constructs: full-length Lrit3 (n = 9), ΔFN3 (n = 9), ΔIG (n = 9), ΔIGFN3 (n = 9), and ΔLRR (n = 9). *E*, quantitative analysis of photopic ffERG b-wave amplitudes across multiple flash intensities for experimental groups described in *panel D*. *F*, cone-isolated b-wave amplitudes from photopic ffERG recordings at 1.4 log cd s/m^2^ comparing all treatment groups from *panel D*. Data are presented as mean ± SEM. One-Way ANOVA with Šídák's multiple comparisons test. FN3, fibronectin type III; LRR, leucine-rich repeat; rAAV8, recombinant adeno-associated virus type 8; RHO, rhodopsin.

Signaling between photoreceptors and DBCs can be assessed using the electroretinogram (ERG). The a-wave represents photoreceptor function and the b-wave the response of DBCs. When measured under scotopic or photopic conditions, it provides functional information about the DBC signalplex in rod and cone DBCs, respectively. The IHC above showed that expression of only the full-length LRIT3 and the ΔFN3 construct restored TRPM1 expression to the dendrites of rod and cone DBCs. This would predict that only these two treatments would restore function. To address this question, treated animals were assessed using the amplitude of the ERG b-wave determined under scotopic and photopic conditions. We measured the scotopic ERG b-wave to assess rod DBC function using flash intensities starting below the cone threshold and extending into the mesopic range, where both rods and cones contribute to the response. We also measured the photopic, cone only, response by saturating the rod response prior to delivery of high intensity flashes. From these data, we extracted the ERG b-wave amplitudes for rod-isolated (−3.6 log cd s/m^2^) and cone-isolated (1.4 log cd s/m^2^) responses for control and treated animals (Fig. 9). Example rod-isolated waveforms of *Lrit3*^*−/−*^ animals treated with the RHO driven LRIT3 constructs (Fig. 9*A*) and summary data for the full flash series (Fig. 9, *B* and *C*) show that only the full-length LRIT3 and ΔFN3 constructs restored a response. To examine the function of the LRIT3 domains on cone photoreceptor function, we quantified the photopic ERG b-wave. Examples of photopic responses in a single eye treated with each construct are shown (Fig. 9*D*), as well as the results from the full photopic flash series for all eyes (Fig. 9, *E* and *F*). These data show that the cone DBC signalplex is only functional when *Lrit3*^*−/−*^ retinas are treated with either the full-length LRIT3 or the ΔFN3 construct.

A summary of the IHC and functional data for all the deletion constructs is presented in Table 1. These data show that the FN3 domain is not required for trafficking in photoreceptors or assembly of a functional signalplex on all DBCs. In both rods and cones, the IG domain is not required for trafficking of the protein to the photoreceptor synapse, as indicated by IHC, but is insufficient to restore a functional signalplex on DBCs. The IG domain is not required for Nyctalopin expression on DBC dendrites; however, it is necessary for proper TRPM1 localization. Finally, the LRR domain is not required for trafficking to the synapse in rods but may be required in cone synapses.

**Table 1:** Function of LRIT3 domains

Lost in Lr*it3*^*−/−*^	RODs Nyctalopin, TRPM1			CONEs Nyctalopin, TRPM1,mGluR6,GPR179		
Construct	Traffic	Restored	Function	Traffic	Restored	Function
Control	Y	All present	Y	Y	All present	Y
*Lrit3*^*−/−*^	N	Nyc, TRP lost	N	N	All lost	N
ΔFN3	Y	Nyc, TRP	Y	Y	Nyc, M6, TRP, GPR	Y
ΔIG	Y	Nyc	N	Y	Nyc, M6,GPR	N
ΔIGFN3	Y	Nyc	N	Y	Nyc, M6,GPR	N
ΔLRR	Y	None	N	N	na	na

FN3, fibronectin type III; mGluR6, metabotropic glutamate receptor 6; Nyc, Nyctalopin, M6, mGluR6, TRP, TRPM1, GPR, GPR179.

## Discussion

We previously reported that LRIT3 is expressed presynaptically at both rod and cone terminals, where it transsynaptically organizes the postsynaptic glutamate signaling complex on depolarizing bipolar cells (17, 21). The absence of LRIT3 leads to differential effects on the rod and cone DBC signalplexes that include mGluR6, GPR179, Nyctalopin, and TRPM1. We hypothesized that these differential effects may be mediated by the individual protein domains of LRIT3, which interact with its postsynaptic partners. In this study, we investigated the specific functions of LRIT3 extracellular domains, FN3, IG, and LRR. Frequently, such experiments are performed in cultured cells because more manipulations are possible; however, the results do not always reflect the *in vivo* situation. We demonstrated this in a previous study: *in vitro*, RGS7 and RGS11 interacted with GPR179, whereas *in vivo*, only RGS7 showed this interaction (25). Therefore, in this study, we chose to use rAAVs to express various LRIT3 constructs *in vivo*. This allowed us to examine trafficking and functional outcome in the normal *in vivo* setting, namely, rods and cones.

LRIT3 is a single-pass transmembrane protein with three extracellular domains. The FN3 domain is most proximal to the membrane, followed by the IG and LRR domains. FN3 domains involve protein-protein interaction that are widely distributed in mammalian cell adhesion proteins. LRIT3 mutants lacking the FN3 domain traffic and localize normally at both rod and cone terminals and restore the DBC function as measured by the ERG b-wave. Included in the ΔFN3 construct is a 142 amino acid region (aa 341–482) that does not contain any known domain. Therefore, the FN3 domain and this linker region are not required for assembly or function of the postsynaptic DBC signalplex. The LRIT3 FN3/linker domain deleted in our tested construct is highly conserved across all mammals, suggesting that it plays a crucial role. One possibility is to position other proteins involved in glutamate dynamics that are expressed on photoreceptor terminals and are not assayed by the ERG. Evidence for this possibility is that in *Lrit3*^*−/−*^ mice, there is reduced excitatory input to OFF bipolar cells, suggesting less glutamate reaches the flat synapses between cones and OFF BC dendrites. Further studies are needed to identify these potential interacting proteins.

The IG domain is located between the FN3 and LRR domains in LRIT3, and constructs lacking either the IG domain or both the IG and FN3 domains have a similar impact on DBC signalplex assembly, namely, loss of TRPM1 from the signalplex of all DBCs. In the absence of the IG domain, Nyctalopin is localized correctly in both rods and cones, as are mGluR6 and GPR179 in cones. These data show that the LRIT3 LRR domain is required for mGluR6 localization to cone DBC dendrites, but not for its localization to rod BC dendrites. Instead, in rod BCs, the localization of mGluR6 is dependent on interactions with ELFN1 (14), mediated by glycosylation of the mGluR6 glutamate binding domain (26, 27). The transsynaptic organization of mGluR6 and GPR179 in cone DBCs is complex and requires both LRIT3 and ELFN2 expression, because in either ELFN1/2 or LRIT3 knockout mice, mGluR6 and GPR179 are absent (15, 19, 21, 28). Why the expression of ELFN1 and/or 2 in cones is insufficient to localize mGluR6 and GPR179, as it does in rod BCs, is unclear; however, this indicates that the mechanisms involved are different in rods and cones.

In previous studies, we reported that insertion of TRPM1 into the dendrites of DBCs, both rod and cone, required Nyctalopin (11), and that TRPM1 and Nyctalopin interact directly (11, 29). However, we now show that expressing the ΔIG or ΔIGFN3 construct inserts Nyctalopin into the DBC membranes, but this is insufficient to restore TRPM1 localization. Thus, Nyctalopin is necessary, but not sufficient, for normal positioning and function of TRPM1 in DBCs. Rather, both Nyctalopin and LRIT3 are required.

The LRR domain in LRR-containing proteins is involved in homomeric, heteromeric, or transsynaptic protein-protein interactions (30). Interaction between Nyctalopin and LRIT3 has been observed in a heterologous expression system (13). In the absence of the LRR domain, the mutant LRIT3 protein traffics normally to rod terminals. Still, it fails to restore Nyctalopin and TRPM1 localization. This may indicate that the LRR domain of LRIT3 interacts transsynaptically with the LRR domain of Nyctalopin. Alternatively, the LRR domain may be required to form homodimers, which then interact with their postsynaptic partner, Nyctalopin, as is the case for ELFNs (31).

The trafficking of synaptic proteins to both presynaptic and postsynaptic locations is essential for proper function. This involves processing through the endoplasmic reticulum, then the Golgi, before being inserted into the membrane, where transsynaptic interactions occur, stabilizing the signaling complexes. These processes require specific domains, including the signal sequence, to ensure trafficking to the correct location. For LRIT3, the requirements for proper trafficking of rods and cones differ. In rods, only the intracellular domain is required, as shown by the trafficking of all constructs with the various extracellular domains deleted. In contrast, in cones, both the intracellular amino acids and the extracellular LRR domain are required. It is possible that the Myc tag we added to the ΔLRR construct interfered with intracellular processing and trafficking. Although this cannot be excluded, the insertion of BioID or APEX proteins does not interfere with function. For ELFN1 and 2, their LRR domains interact to form homodimers or heterodimers before they are inserted into the membrane, whereupon they interact with group III mGluRs (31). Whether a similar mechanism for LRIT3 is involved in cones remains to be determined. Another caveat is that our studies were conducted in adult mice, whereas normal synapse formation occurs during early postnatal development; however, the finding that LRIT3 expression in adult mice fully restores function mitigates this concern to some extent, and we found rAAV8 RHO::ΔFN3 treatment of P5 *Lrit3*^*−/−*^ pups rescued function (Fig. S4).

Our study is the first to elucidate the *in vivo* roles of LRIT3 domains in synaptic function and protein assembly in the retina, reveals that each domain has a distinct function at both rod and cone metabotropic synapses, although some are still unknown. The LRR domain is essential for the localization of Nyctalopin and TRPM1 at rod synapses and maybe involved in intracellular processing in cones. Our findings did not detect any involvement of the FN3 domain in signalplex assembly; however, both the IG and LRR domains are vital for ON pathway signaling. Future studies will be essential to elucidate the role of the FN3 domain in synapse function and why, in cones, both LRIT3 and ELFN2 are required for postsynaptic DBC signalplex assembly.

## Experimental procedures

### Animals

All procedures were conducted following the Society for Neuroscience policies on the use of animals in research and the University of Louisville Institutional Animal Care and Use Committee guidelines. Animals were housed in the University's Association for the Assessment and Accreditation of Laboratory Animal Care, International-approved facility under a 12-h light/dark cycle. The phenotype of the *Lrit3*^*−/−*^ mouse line used in this study has been previously described (13, 17, 21).

Both male and female *Lrit3*^*−/−*^ and C57BL/6J (WT) mice were used throughout the study. Mice were anesthetized with a ketamine/xylazine solution (118/11 mg/kg, respectively) diluted in normal Ringer’s solution before subretinal injections and ERG recordings. For enucleation of the eyes, mice were euthanized by CO_2_ exposure in accordance with American Veterinary Medical Association guidelines. Data from mice with gross retinal damage from the injection procedure were excluded from the analyses.

### Antibodies

Antibodies used to label rod and cone synaptic proteins in the retina have been previously described (13, 17, 21) and are listed in Table 2. All antibodies were used at a dilution of 1:1000 and were validated for specificity by testing sections from the respective knockout mouse retinas. A GFP antibody was used to detect Nyctalopin-EYFP (24).

**Table 2:** Antibodies used

Antibodies	Source	Identifier
Guinea pig anti-LRIT3	Gregg Lab (13)	N/A
Goat anti-mGluR6	Gregg Lab (17)	N/A
Rabbit anti-mCAR	Sheryl Craft (38)	N/A
Rabbit anti-TRPM1	Gregg Lab (13)	N/A
Chicken anti-GFP	Invitrogen	Cat# A10262
Sheep anti-GPR179	Gregg Lab (39)	N/A
Donkey anti-sheep IgG-AlexaFluor 488	Life Technologies	Cat# A111015
Donkey anti-rabbit IgG-AlexaFluor 647	Life Technologies	Cat# A31573
Donkey anti-guinea pig IgG-Cy3	Millipore	Cat# AP193C
Donkey anti-rabbit IgG-AlexaFluor 488	Life Technologies	Cat# A21206
Donkey anti-goat IgG-AlexaFluor 647	Life Technologies	Cat# A32849
Donkey anti-chicken IgG-AlexaFluor 488	Jackson Immuno Research Laboratories	Cat# 703–545–155; RRID: AB_2340375

mGluR6, metabotropic glutamate receptor 6; IgG, immunoglobulin G.

### Retina preparation for immunohistochemistry

Retina sections and whole mounts were prepared following the methods described previously (17, 21). Briefly, mice were euthanized by CO_2_ inhalation followed by cervical dislocation. Eyes were enucleated, and the cornea and lens were removed. Retinas were dissected in PBS (pH 7.4) and fixed for 15 to 30 min in 4% paraformaldehyde diluted in phosphate buffer (0.1 M PB). After fixation, retinas were washed three times in PBS for 10 min each, cryoprotected in a graded series of sucrose solutions (5%, 10%, and 15% in 0.1 M PB) for 1 h at room temperature and then overnight at 4 °C in 20% sucrose in 0.1 M PB. Retinas were incubated in a mixture of Optimal Cutting Temperature compound and 20% sucrose (2:1) for 1 h prior to embedding. Cryoprotected retinas were frozen, and 18 μm transverse sections cut using a cryostat (Leica Biosystems). Sections were mounted on Superfrost Plus slides (Thermo Fisher Scientific) and stored at −80 °C. IHC methods have been previously described (13, 32). Retina sections and whole mounts were imaged using an FV4000 Confocal Microscope (Olympus). Contrast and brightness adjustments were made using Fluoview Software (Olympus) or Photoshop (Adobe Systems).

### rAAV production and injection

To identify and annotate domains of LRIT3, we used the online SMART application (Simple Modular Architecture Research Tool (33), and AlphaFold2 (34). WT and deletion mutants were from a mouse cDNA, Accession # KF954709, containing a c.55G > T substitution (sequence of LRIT3 ORFs is provided as Table S1. Deletion mutants were cloned into the pcDNA3.1 vector. To express WT and deletion mutant LRIT3 proteins (ΔFN3, ΔIG, ΔIGFN3, and ΔLRR) in rods and cones, we employed rAAV8s with gene expression controlled by the RHO and GNAT2 promoters, respectively (17, 21). The LRIT3 expression constructs were packaged into the rAAV8 capsid by VectorBuilder. The rAAVs expressing WT or deletion mutants of LRIT3 under the RHO promoter are designated as rAAV RHO::Lrit3, rAAV RHO::ΔFN3, rAAV RHO::ΔIG, rAAV RHO::ΔIGFN3, and rAAV RHO::LRR. For cone expression constructs, the RHO promoter was replaced by the GNAT2 promoter (23).

rAAV injections were performed by introducing 1 μl of the rAAV solution (1 × 10^13^ vg/ml in PBS) into the subretinal space of adult mice (postnatal day 35) using a specialized syringe (www.borghuisinstruments.com). Synaptic protein expression and retinal function were assessed 4 to 8 weeks after rAAV injection.

### Electroretinography

ERG recordings were performed as previously described (13, 35). Mice were dark-adapted overnight to ensure optimal sensitivity. They were then anesthetized with a ketamine/xylazine solution (118/11 mg/kg, respectively) diluted in Ringer’s solution, and prepared for recordings under dim red light to minimize additional light exposure. To prepare the mice, their pupils were dilated using topical applications of 0.625% phenylephrine hydrochloride and 0.25% tropicamide. The corneal surface was anesthetized with 1% proparacaine HCl. A feedback-controlled electric heating pad (TC1000; CWE Inc) was used to maintain body temperature throughout the procedure. A contact lens with a gold electrode (LKC Technologies Inc) was placed on the cornea and moistened with artificial tears (Tears Again; OCuSOFT) to ensure proper electrical contact. Ground and reference needle electrodes were positioned in the tail and on the midline of the forehead, respectively. For the ERG measurements, scotopic responses were recorded in dark-adapted mice to test flashes ranging from −3.6 to 1.4 log cd s/m^2^. Photopic responses were measured with test flashes ranging from −0.8 to 1.4 log cd s/m^2^ after a 5 min light adaptation to a rod-saturating background of 20 cd/m^2^.

### Rod and cone counts

The percentage of rod and cone photoreceptors expressing either WT or mutant LRIT3 in rAAV-treated *Lrit3*^*−/−*^ retinas was quantified from wholemount immunostained images, as previously described (17). To identify all cone photoreceptors, retinas were stained with cone arrestin, while mGluR6 was used to label all rod synapses. LRIT3 staining was employed to mark transfected cells. For each rAAV expression construct, images were captured from three transfected retinas of three different mice using an FV4000 confocal microscope with a 40x objective (NA = 1.4). Five images were taken per retina: one from each of the four quadrants and one from the central retina. These images were analyzed to determine the number of rods and cones expressing LRIT3 constructs. The total number of rod and cone synapses was estimated by counting mGluR6 puncta and cone arrestin, respectively. ImageJ software was used to count the puncta from maximum projections of z-stack whole mount images.

### Immunoblotting

Western blot analysis of LRIT3 deletion constructs was performed as previously described (36, 37). Briefly, rAAV injections were performed in adult mice as described above. Five weeks post injection, mice were euthanized and retinas were isolated and homogenized in lysis buffer (1% Nonidet P-40, 2 mM EDTA (ethylenediaminetetraacetic acid), and 20 mM Hepes (4-(2-hydroxyethyl)-1-piperazineethanesulfonic acid), pH 7.4) supplemented with protease inhibitor cocktail (Sigma-Aldrich) by rotating for 45 min at 4 °C. Samples were centrifuged at 17,000*g* for 20 min at 4 °C to remove cell debris, and the supernatant was collected and protein content quantified using Bradford reagent (Bio-Rad). Protein lysates were analyzed on 4 to 12% NuPAGE gels (Invitrogen), transferred to polyvinylidene fluoride membranes, and blocked with Odyssey blocking buffer. Membranes were incubated with primary antibodies (LRIT3 or MYC) diluted in Odyssey blocking buffer and washed four times with TBS containing 0.1% Tween-20 (TBST). After incubating with IRDye 800CW and IRDye 680CW-conjugated secondary antibodies diluted in Odyssey blocking buffer (LI-COR Biosciences), membranes were washed four times with TBST. Protein bands were visualized by scanning the membranes in an Odyssey Infrared Imaging System (LI-COR Biosciences) using both 700 and 800 nm channels.

### Statistical analysis

Statistical analysis was performed using Prism 10.5.0 (GraphPad Software, Inc). Details of the statistical methods are provided in the text and figure legends. Šídák's *post hoc* tests were applied to adjust for multiple comparisons where appropriate, and the adjusted *p* values are reported. Statistical significance was defined as p_j_ ≤ 0.05.

## Data availability

All data are available on reasonable request.

## Supporting information

This article contains supporting information.

## Conflict of interest

The authors declare that they have no conflicts of interest with the contents of this article.
