# Supplementary material for: Domain-specific functions of LRIT3 in synaptic assembly and retinal signal transmission
Source: J Biol Chem. 2026 Apr 27;302(6):113097. doi: 10.1016/j.jbc.2026.113097 (PMC13224074; doi:10.1016/j.jbc.2026.113097)
Supplement: Table S1 [file mmc1.docx]

Supplementary Table 1 cDNA sequences of ORFs for each deletion construct

**LRIT3 Wildtype** ATGTGGCTCTCGGCCTGCCTGTGCCTTGTGCTTAGCTTCCTGGGAGGAGTGAACTGTACCTGCCCTTCTCAGTGCTCCTGTGAGTATCACGGCAGACATGACGGCTCAGGATCAAGGTTAGTGTTGTGTAACGACCTGGATATGAACGAAGTCCCAGCAAACTTCCCCGTGGACACCTCGAAGCTTCGCATAGAGAAGACTGTGGTCCGCAGGCTCCCCGCCGAGGCCTTCTACTACCTGGTGGAGCTGCAGTACCTCTGGCTGGCTTACAACTCAGTGGCCAGCATAGAAACCAGTAGCTTCTATAACCTGAGGCAGCTGCACGAGTTGCGTTTGGATGGGAATTCTCTGACCGCTTTCCCTTGGGTGTCTCTGCTGGACATGCCCCACCTGAGGACGCTGGACTTACACAATAACAGAATAGCCAGTGTGCCAAACGAGGCGGTCAGATATCTGAGGAACCTCACCTGCTTGGACTTGTCGAGCAACCGACTGACCACGCTGCCACCAGATTTCCTAGACAGCTGGTCTCATTTAGCCGTGACACCGTCTAGAAGCCCGGACTTTCCACCGAGAAGAATTATTCTTGGTTTGCAGGACAACCCCTGGTTCTGTGACTGTCACATTTCCAAGGTGATCGAGCTGTCGAAAGTCACCGACCACGCTGTTGTTCTTCTTGATCCTCTGATGGTCTGCAGTGAACCCGAGCGCTTCCAAGGAATCTTGTTCCAGAGGGTAGAGTTGGAAAAGTGTCTGAAGCCGTCCGTGATGATGTCAGCTACCAAAATCACATCTGCTCTGGGTAGTAATGTTCTGCTGAGATGTGATGCCAAGGGTCACCCCACCCCACAGCTGACGTGGACCAGATCCGACGGCTCCACAGTTAACTATACAGTAATTCAGGAGTCTCCAGGAGAAGGCATCAGATGGTCCATCATAAGCTTGACCAGCATCTCTCACAAGGATGCTGGGGATTACAGGTGTAAAGCCAAAAATCTGGCAGGGATTTCGGAAGCTGTGGTTACTGTGACAGTTGTTGGTGGTGTTACGACCACCCTATCACCAGACAGTTCAGAAAGAAGTCCTGGAGAGCCCCCTGAGCAGCATCCCCAGCCAGGATTAGGAGGATCAACACCTCCATCTAAATCCTGGTTATCACCCGGGCTCACTTCCGCTCCTTCCTACCCCACCCCCTCAGCAGCTCTCTATACATCTACGTGGTCCCCTCCTCCCTCCTCCTTGCCTCCCATCTTCTCAGCTGCTTCTGCAACCACCAGTGTACAAACCAGCATCTCAGGACGCACCGCCAGGACTAGCCACCAGCCACCCCTGCTCCACCCCGGTGGGAAAAGCAATGCGAAGATAGAGAAGAACGGAAGGAAGTTTCCTCCGCTCAGCGCAAGTAAGAAAGAAGAGTTGGCGTTGTTGGATCAGGCAGCGCCAATGGAAACAAACGTCACTATCAAAGACCTCAGGGTGGCCCGTGAAACCGGCGTGAGTGTGACTCTGATGTGGAACAGCAGCAGCAGCACACAAGAGTCCTCTGTGACTGTGCTGTATTCTAAGTACGGTGAGAAGGACCTGCTGTTAGTGAATGCAGACGACTATGGCAAGAACCAGGCAACCATAAATGGCCTAGAGCCCGGAAGTCAGTATGTGGCATGTGTCTGTCCAAAAGGAGTGGGTCCCCGGGAGGATCTGTGTATCACCTTTTCTACCAACAGAGTTGAGGGGCGTGGCTCACAGTGGTCATTGCTCCTCGTGGTGACCAGTACTGCCTGTGTTATAGTCGTGCCCCTAATTTGTTTCTTATTATATAAAGTCTGCAAATTGCAATGCACATCGGACCCTTTCTGGGAAGAGGATTTGTCAAAAGAGACATATATCCAATTTGAGACCCTGTCACCCAGGTCACAGAGTATAGGGGAGCTCTGGACCCGGAGGCACAGAGACGATGGCGAGAGGTTGCTGCTATGCTCCCAGTCAAGTGTGGACTCCCAGATGAATCTTAAGAGCGATGGCTGTAGGACGGAGTATTATGGCTGA

**ΔLRR** ATGTGGCTCTCGGCCTGCCTGTGCCTTGTGCTTAGCTTCCTGGGAGGAGTGAACTGCACAGAGCAGAAACTCATCTCAGAAGAGGATCTGGCAGCAAATGATCTGGATTACAAGGATGACGACGATAAGGATACCAAAATCACATCTGCTCTGGGTAGTAATGTTCTGCTGAGATGTGATGCCAAGGGTCACCCCACCCCACAGCTGACGTGGACCAGATCCGACGGCTCCACAGTTAACTATACAGTAATTCAGGAGTCTCCAGGAGAAGGCATCAGATGGTCCATCATAAGCTTGACCAGCATCTCTCACAAGGATGCTGGGGATTACAGGTGTAAAGCCAAAAATCTGGCAGGGATTTCGGAAGCTGTGGTTACTGTGACAGTTGTTGGTGGTGTTACGACCACCCTATCACCAGACAGTTCAGAAAGAAGTCCTGGAGAGCCCCCTGAGCAGCATCCCCAGCCAGGATTAGGAGGATCAACACCTCCATCTAAATCCTGGTTATCACCCGGGCTCACTTCCGCTCCTTCCTACCCCACCCCCTCAGCAGCTCTCTATACATCTACGTGGTCCCCTCCTCCCTCCTCCTTGCCTCCCATCTTCTCAGCTGCTTCTGCAACCACCAGTGTACAAACCAGCATCTCAGGACGCACCGCCAGGACTAGCCACCAGCCACCCCTGCTCCACCCCGGTGGGAAAAGCAATGCGAAGATAGAGAAGAACGGAAGGAAGTTTCCTCCGCTCAGCGCAAGTAAGAAAGAAGAGTTGGCGTTGTTGGATCAGGCAGCGCCAATGGAAACAAACGTCACTATCAAAGACCTCAGGGTGGCCCGTGAAACCGGCGTGAGTGTGACTCTGATGTGGAACAGCAGCAGCAGCACACAAGAGTCCTCTGTGACTGTGCTGTATTCTAAGTACGGTGAGAAGGACCTGCTGTTAGTGAATGCAGACGACTATGGCAAGAACCAGGCAACCATAAATGGCCTAGAGCCCGGAAGTCAGTATGTGGCATGTGTCTGTCCAAAAGGAGTGGGTCCCCGGGAGGATCTGTGTATCACCTTTTCTACCAACAGAGTTGAGGGGCGTGGCTCACAGTGGTCATTGCTCCTCGTGGTGACCAGTACTGCCTGTGTTATAGTCGTGCCCCTAATTTGTTTCTTATTATATAAAGTCTGCAAATTGCAATGCACATCGGACCCTTTCTGGGAAGAGGATTTGTCAAAAGAGACATATATCCAATTTGAGACCCTGTCACCCAGGTCACAGAGTATAGGGGAGCTCTGGACCCGGAGGCACAGAGACGATGGCGAGAGGTTGCTGCTATGCTCCCAGTCAAGTGTGGACTCCCAGATGAATCTTAAGAGCGATGGCTGTAGGACGGAGTATTATGGCTGA

**ΔIG** ATGTGGCTCTCGGCCTGCCTGTGCCTTGTGCTTAGCTTCCTGGGAGGAGTGAACTGCACGTGCCCTTCTCAGTGCTCCTGTGAGTATCACGGCAGACATGACGGCTCAGGATCAAGGTTAGTGTTGTGTAACGACCTGGATATGAACGAAGTCCCAGCAAACTTCCCCGTGGACACCTCGAAGCTTCGCATAGAGAAGACTGTGGTCCGCAGGCTCCCCGCCGAGGCCTTCTACTACCTGGTGGAGCTGCAGTACCTCTGGCTGGCTTACAACTCAGTGGCCAGCATAGAAACCAGTAGCTTCTATAACCTGAGGCAGCTGCACGAGTTGCGTTTGGATGGGAATTCTCTGACCGCTTTCCCTTGGGTGTCTCTGCTGGACATGCCCCACCTGAGGACGCTGGACTTACACAATAACAGAATAGCCAGTGTGCCAAACGAGGCGGTCAGATATCTGAGGAACCTCACCTGCTTGGACTTGTCGAGCAACCGACTGACCACGCTGCCACCAGATTTCCTAGACAGCTGGTCTCATTTAGCCGTGACACCGTCTAGAAGCCCGGACTTTCCACCGAGAAGAATTATTCTTGGTTTGCAGGACAACCCCTGGTTCTGTGACTGTCACATTTCCAAGGTGATCGAGCTGTCGAAAGTCACCGACCACGCTGTTGTTCTTCTTGATCCTCTGATGGTCTGCAGTGAACCCGAGCGCTTCCAAGGAATCTTGTTCCAGAGGGTAGAGTTGGAAAAGTGTCTGAAGCCGTCCGTGATGGCGCCAATGGAAACAAACGTCACTATCAAAGACCTCAGGGTGGCCCGTGAAACCGGCGTGAGTGTGACTCTGATGTGGAACAGCAGCAGCAGCACACAAGAGTCCTCTGTGACTGTGCTGTATTCTAAGTACGGTGAGAAGGACCTGCTGTTAGTGAATGCAGACGACTATGGCAAGAACCAGGCAACCATAAATGGCCTAGAGCCCGGAAGTCAGTATGTGGCATGTGTCTGTCCAAAAGGAGTGGGTCCCCGGGAGGATCTGTGTATCACCTTTTCTACCAACAGAGTTGAGGGGCGTGGCTCACAGTGGTCATTGCTCCTCGTGGTGACCAGTACTGCCTGTGTTATAGTCGTGCCCCTAATTTGTTTCTTATTATATAAAGTCTGCAAATTGCAATGCACATCGGACCCTTTCTGGGAAGAGGATTTGTCAAAAGAGACATATATCCAATTTGAGACCCTGTCACCCAGGTCACAGAGTATAGGGGAGCTCTGGACCCGGAGGCACAGAGACGATGGCGAGAGGTTGCTGCTATGCTCCCAGTCAAGTGTGGACTCCCAGATGAATCTTAAGAGCGATGGCTGTAGGACGGAGTATTATGGCTGA

**ΔFN3** ATGTGGCTCTCGGCCTGCCTGTGCCTTGTGCTTAGCTTCCTGGGAGGAGTGAACTGCACGTGCCCTTCTCAGTGCTCCTGTGAGTATCACGGCAGACATGACGGCTCAGGATCAAGGTTAGTGTTGTGTAACGACCTGGATATGAACGAAGTCCCAGCAAACTTCCCCGTGGACACCTCGAAGCTTCGCATAGAGAAGACTGTGGTCCGCAGGCTCCCCGCCGAGGCCTTCTACTACCTGGTGGAGCTGCAGTACCTCTGGCTGGCTTACAACTCAGTGGCCAGCATAGAAACCAGTAGCTTCTATAACCTGAGGCAGCTGCACGAGTTGCGTTTGGATGGGAATTCTCTGACCGCTTTCCCTTGGGTGTCTCTGCTGGACATGCCCCACCTGAGGACGCTGGACTTACACAATAACAGAATAGCCAGTGTGCCAAACGAGGCGGTCAGATATCTGAGGAACCTCACCTGCTTGGACTTGTCGAGCAACCGACTGACCACGCTGCCACCAGATTTCCTAGACAGCTGGTCTCATTTAGCCGTGACACCGTCTAGAAGCCCGGACTTTCCACCGAGAAGAATTATTCTTGGTTTGCAGGACAACCCCTGGTTCTGTGACTGTCACATTTCCAAGGTGATCGAGCTGTCGAAAGTCACCGACCACGCTGTTGTTCTTCTTGATCCTCTGATGGTCTGCAGTGAACCCGAGCGCTTCCAAGGAATCTTGTTCCAGAGGGTAGAGTTGGAAAAGTGTCTGAAGCCGTCCGTGATGATGTCAGCTACCAAAATCACATCTGCTCTGGGTAGTAATGTTCTGCTGAGATGTGATGCCAAGGGTCACCCCACCCCACAGCTGACGTGGACCAGATCCGACGGCTCCACAGTTAACTATACAGTAATTCAGGAGTCTCCAGGAGAAGGCATCAGATGGTCCATCATAAGCTTGACCAGCATCTCTCACAAGGATGCTGGGGATTACAGGTGTAAAGCCAAAAATCTGGCAGGGATTTCGGAAGCTGTGATCACCTTTTCTACCAACAGAGTTGAGGGGCGTGGCTCACAGTGGTCATTGCTCCTCGTGGTGACCAGTACTGCCTGTGTTATAGTCGTGCCCCTAATTTGTTTCTTATTATATAAAGTCTGCAAATTGCAATGCACATCGGACCCTTTCTGGGAAGAGGATTTGTCAAAAGAGACATATATCCAATTTGAGACCCTGTCACCCAGGTCACAGAGTATAGGGGAGCTCTGGACCCGGAGGCACAGAGACGATGGCGAGAGGTTGCTGCTATGCTCCCAGTCAAGTGTGGACTCCCAGATGAATCTTAAGAGCGATGGCTGTAGGACGGAGTATTATGGCTGA

**ΔIGFN3** ATGTGGCTCTCGGCCTGCCTGTGCCTTGTGCTTAGCTTCCTGGGAGGAGTGAACTGCACGTGCCCTTCTCAGTGCTCCTGTGAGTATCACGGCAGACATGACGGCTCAGGATCAAGGTTAGTGTTGTGTAACGACCTGGATATGAACGAAGTCCCAGCAAACTTCCCCGTGGACACCTCGAAGCTTCGCATAGAGAAGACTGTGGTCCGCAGGCTCCCCGCCGAGGCCTTCTACTACCTGGTGGAGCTGCAGTACCTCTGGCTGGCTTACAACTCAGTGGCCAGCATAGAAACCAGTAGCTTCTATAACCTGAGGCAGCTGCACGAGTTGCGTTTGGATGGGAATTCTCTGACCGCTTTCCCTTGGGTGTCTCTGCTGGACATGCCCCACCTGAGGACGCTGGACTTACACAATAACAGAATAGCCAGTGTGCCAAACGAGGCGGTCAGATATCTGAGGAACCTCACCTGCTTGGACTTGTCGAGCAACCGACTGACCACGCTGCCACCAGATTTCCTAGACAGCTGGTCTCATTTAGCCGTGACACCGTCTAGAAGCCCGGACTTTCCACCGAGAAGAATTATTCTTGGTTTGCAGGACAACCCCTGGTTCTGTGACTGTCACATTTCCAAGGTGATCGAGCTGTCGAAAGTCACCGACCACGCTGTTGTTCTTCTTGATCCTCTGATGGTCTGCAGTGAACCCGAGCGCTTCCAAGGAATCTTGTTCCAGAGGGTAGAGTTGGAAAAGTGTCTGAAGCCGTCCGTGATGATCACCTTTTCTACCAACAGAGTTGAGGGGCGTGGCTCACAGTGGTCATTGCTCCTCGTGGTGACCAGTACTGCCTGTGTTATAGTCGTGCCCCTAATTTGTTTCTTATTATATAAAGTCTGCAAATTGCAATGCACATCGGACCCTTTCTGGGAAGAGGATTTGTCAAAAGAGACATATATCCAATTTGAGACCCTGTCACCCAGGTCACAGAGTATAGGGGAGCTCTGGACCCGGAGGCACAGAGACGATGGCGAGAGGTTGCTGCTATGCTCCCAGTCAAGTGTGGACTCCCAGATGAATCTTAAGAGCGATGGCTGTAGGACGGAGTATTATGGCTGA
