# Supplementary material for: Domain-specific functions of LRIT3 in synaptic assembly and retinal signal transmission
Source: J Biol Chem. 2026 Apr 27;302(6):113097. doi: 10.1016/j.jbc.2026.113097 (PMC13224074; doi:10.1016/j.jbc.2026.113097)
Supplement: Figures S1–S4 [file mmc2.pdf]

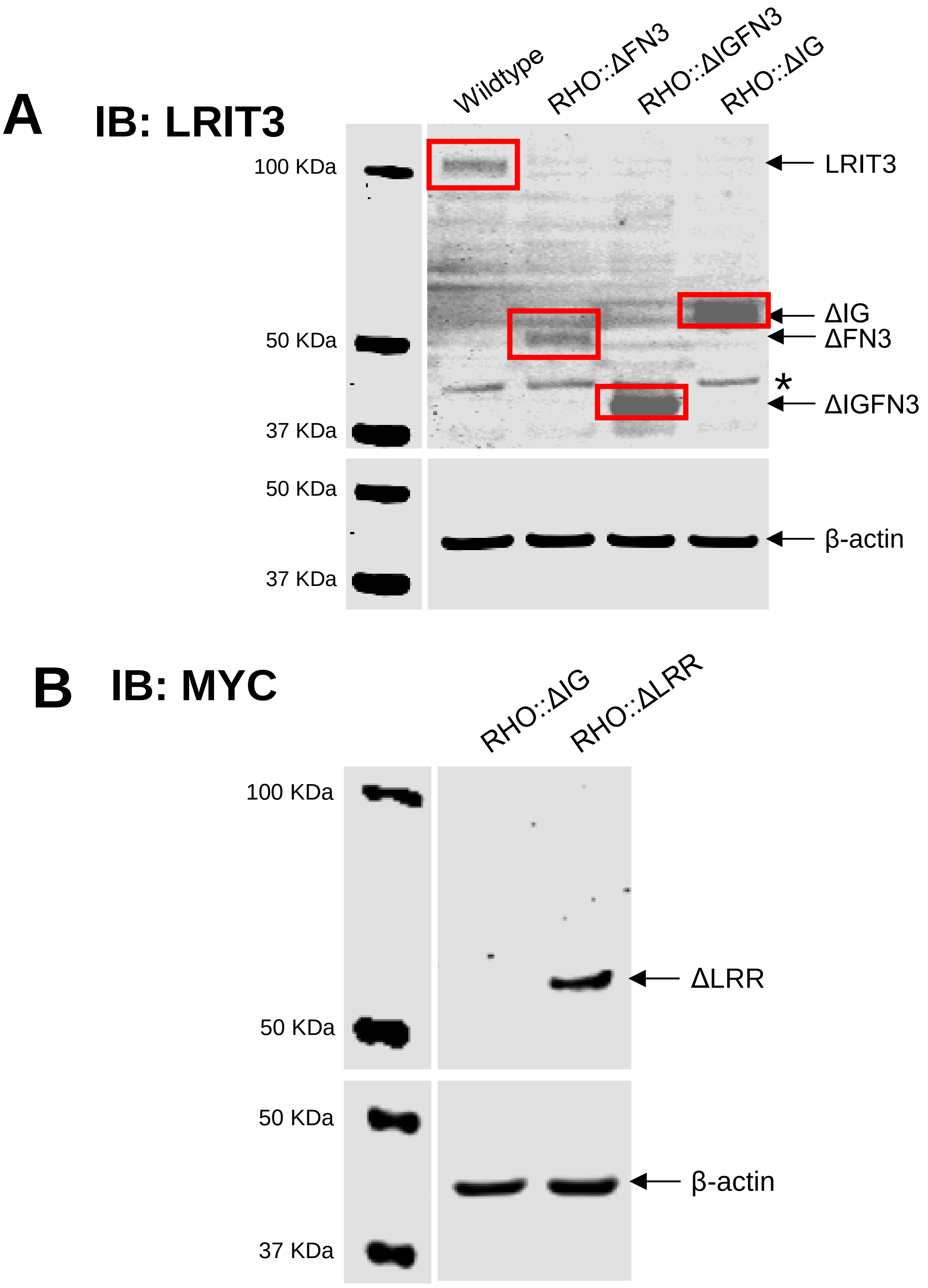

Supplementary Figure 1. Expression of LRIT3 RHO driven deletion constructs in the mouse retina. (A) Western blot analysis of lysates of retinas treated with RHO::ΔFN3, RHO::ΔIG and RHO::ΔIGFN3 using LRIT3 antibody. (B) Western blot analysis of lysates of retinas treated with RHO:: ΔLRR using MYC antibody. All LRIT3 mutant constructs are expressed at the expected molecular size. β-actin was used as a loading control.

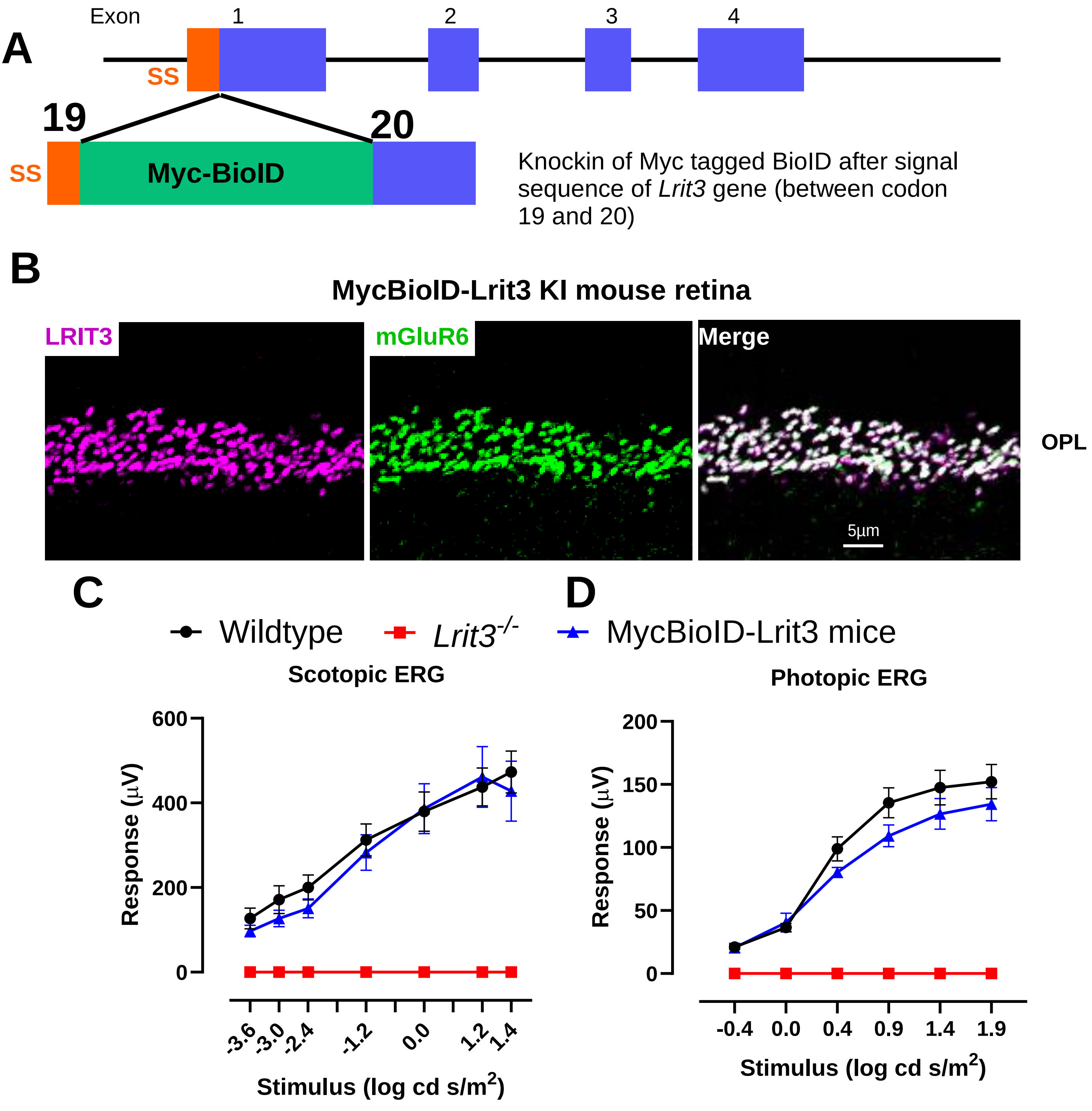

Supplementary Figure 2. Knockin of Myc-BioID into LRIT3 in mouse has normal function. A, Schematic of BioID knockin mouse. The BioID cDNA sequence was inserted between codons 19 and 20 of the *Lrit3* gene. B, IHC with LRIT3 and mGluR6 antibodies shows puncta representing rod and cone terminals. C,D, Amplitude of the scotopic (C) and photopic (D) ERG b-wave in LRIT3-BioID-KI mice is similar to wildtype.

These data show that insertion of the BioID gene did not interfere with synaptic fucntion.

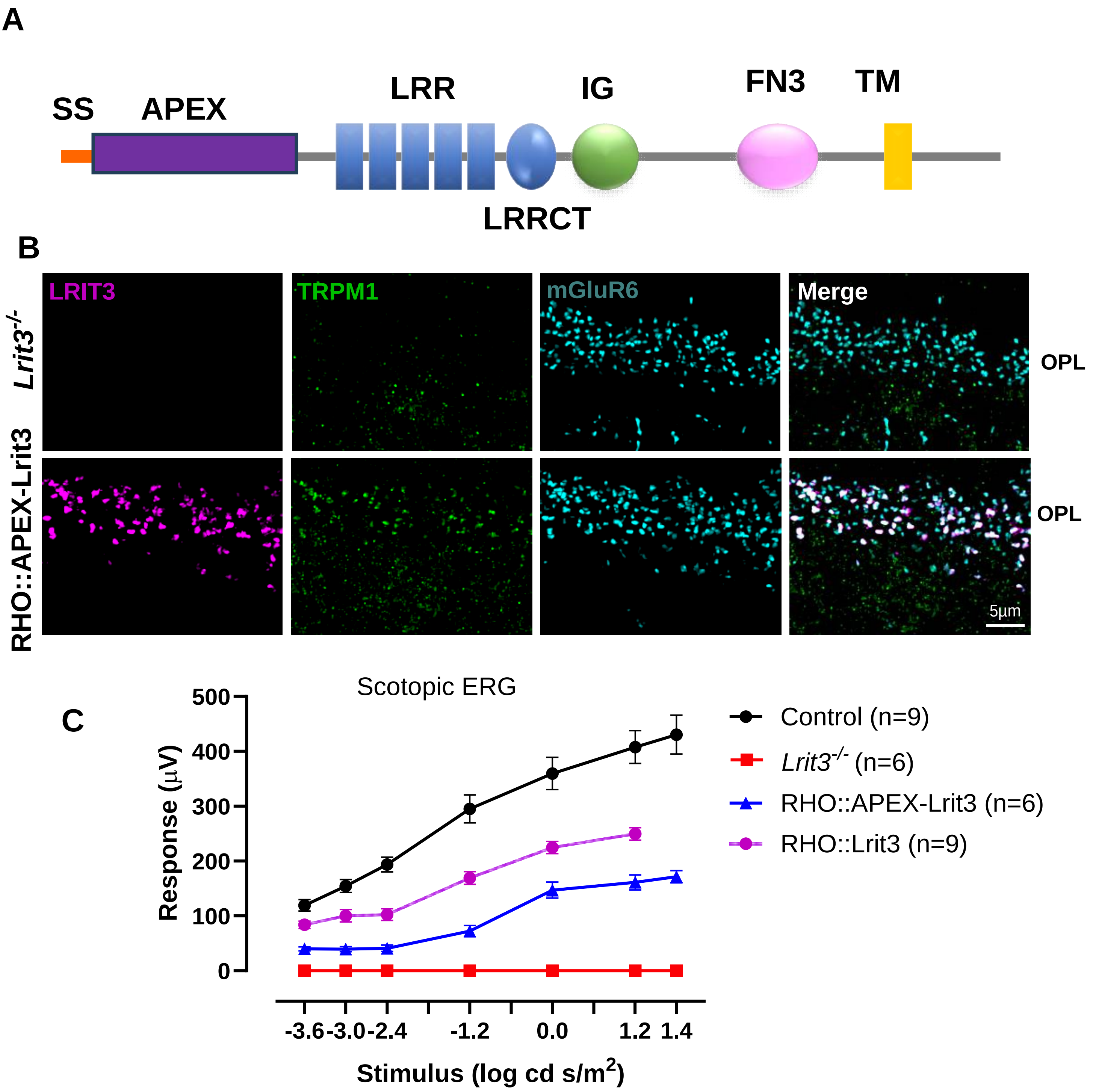

Supplementary Figure 3. rAAV RHO::APEX-LRIT3 expression in *Lrit3*$^{-/-}$ mice. (A) Schematic of the rAAV vector expressing APEX-LRIT3 in rods. APEX was inserted between codons 19 and 20 of LRIT3 (B) IHC with LRIT3, TRPM1, and mGluR6 antibodies of *Lrit3*$^{-/-}$ retinas treated with APEX::Lrit3 shows restoration of LRIT3 and TRPM1 at rod synapses of treated retinas. (C) Amplitude of the scotopic ERG b-wave in control and *Lrit3*$^{-/-}$ retinas treated with RHO:Lrit3 or RHO::Apex-Lrit3.Mean±SEM.

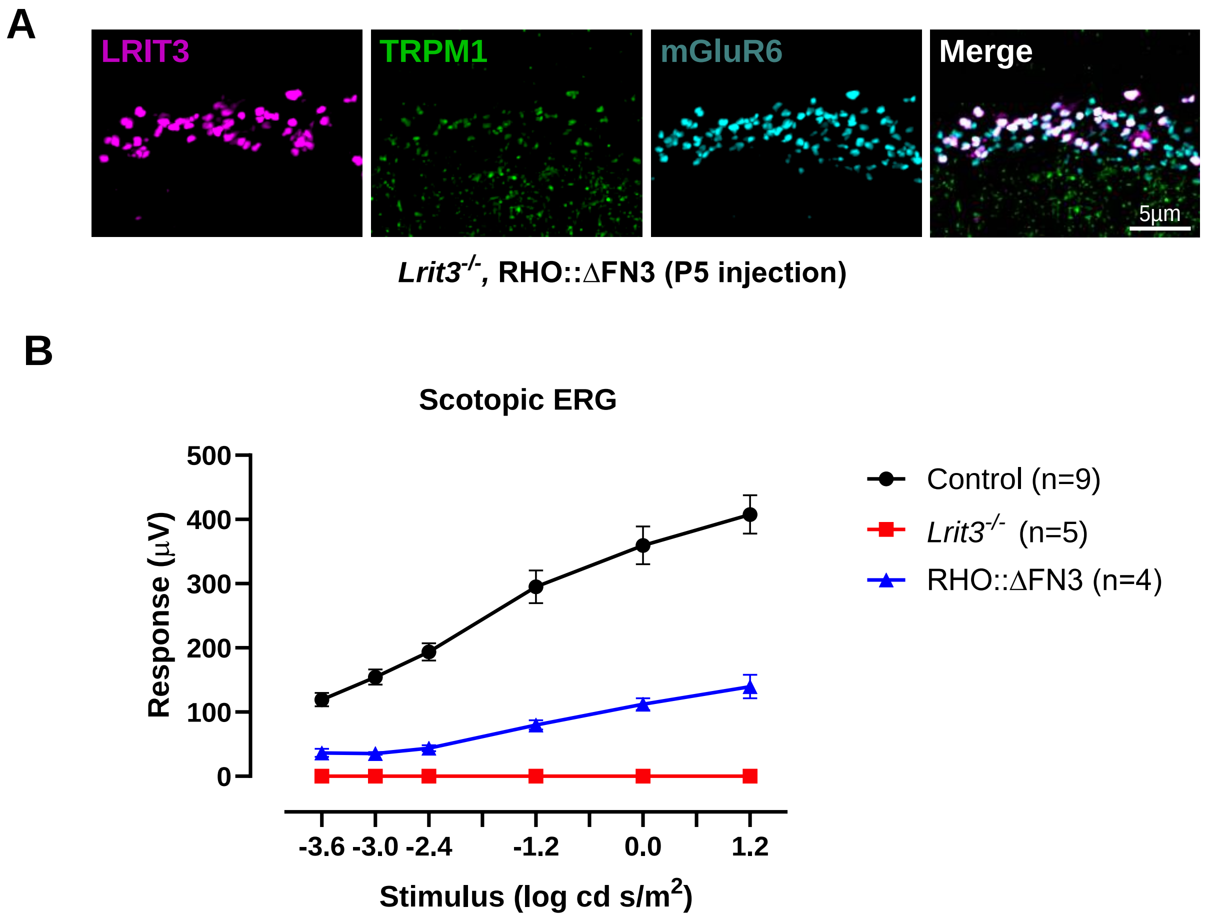

Supplementary Figure 4. rAAV RHO::ΔFN3 expression in *Lrit3*$^{-/-}$ pups treated at P5 restores function. rAAV RHO:: ΔFN3 was injected in *Lrit3*$^{-/-}$ pups at postnatal day 5 (P5), and ERG and IHC analysis was performed 5 weeks after injections. (A) IHC with LRIT3, TRPM1, and mGluR6 antibodies shows restoration of LRIT3 and TRPM1 at rod synapses of RHO:: ΔFN3 treated retinas. (B) Amplitude of the scotopic ERG b-wave in control and treated *Lrit3*$^{-/-}$ mice.
